# Numerical heat transfer simulation system based on Lagrangian particle mathematical model and SSPH algorithm

**DOI:** 10.1371/journal.pone.0313250

**Published:** 2024-12-31

**Authors:** Dan Fang, Conghui Xiong, Wei Yu

**Affiliations:** School of Public Administration, Chongqing Vocational College of Public Transportation, Chongqing, China; University of Science and Technology of China, CHINA

## Abstract

With the continuous promotion and development of national industrialization, the performance control of precision casting and mechanical structures has become increasingly important. The traditional craftsmanship in the foundry industry relies on experience and trial-and-error method, resulting in high cost and long trial production cycles. A numerical heat transfer simulation system is proposed based on the Lagrangian particle mathematical model and optimized numerical heat transfer simulation algorithm. The simulation results showed that the simulation accuracy of the system was as high as 99.1%. The simulation time was stable at 434 ms, with CPU usage and memory usage controlled at 23% and 18%, respectively, surpassing the comparison system. The calculated results do not show any non-physical oscillations, ensuring the authenticity and reliability of simulation results. The results indicate that the numerical heat transfer simulation system provides an efficient, accurate, and low resource consuming simulation tool for the foundry industry, which promotes the rapid development of the manufacturing industry.

## 1 Introduction

With the rapid development of the manufacturing industry, the casting industry is an important component of the manufacturing industry, which places higher demands on improving product quality, increasing productivity, and controlling cost [1,2]. The casting process involves the flow, solidification, and cooling of liquid metal in complex molds, which directly affects the performance and quality of castings. However, traditional casting processes rely on experience and trial-and-error method, resulting in high production cost and long trial production cycles. To address these issues, some researchers have used computer technology to assist various manufacturing processes in the traditional casting industry [3,4]. In recent years, the numerical heat transfer simulation system has brought revolutionary changes to the casting industry. This system describes liquid metals through particles and uses the Smooth Particle Hydrodynamics (SPH) algorithm to calculate flow and temperature fields [5]. It does not require predefined grids and can accurately simulate complex phenomena such as free surface flow, large deformation, and impact. However, relevant research has to some extent solved the shortcomings of traditional methods that rely on experience and trial-and-error method. Some methods rely on specific software or algorithms, limiting their universality and scalability. At the same time, the computational efficiency of large-scale simulation tasks still needs to be improved, especially when dealing with multi-physics field coupling problems. Therefore, the study innovatively adopts the Lagrangian particle mathematical model and improved SPH algorithm to achieve efficient and accurate numerical heat transfer simulation through steps such as particle problem domain, configuring smooth functions, kernel approximation, and field function calculation. Virtual particles and symbol particles are used as processing boundary conditions. The linked list search method is used to improve computational efficiency, enabling the system to handle large-scale simulation tasks. Finally, a Lagrangian Smooth Particle Hydrodynamics-Smooth Particle Hydrodynamics (LSPH-SSPH) heat transfer simulation system is designed and applied in the actual workpiece casting process to verify the effectiveness of the scheme. It is expected that this scheme can effectively improve the accuracy of the numerical heat transfer simulation system and provide a new tool for the rapid development of the manufacturing industry.

The innovation of the research algorithm is mainly reflected in the following aspects: 1. Linked list search method: Dividing the problem domain into grids that match the size of the particle support domain, the particle neighborhood is quickly located, significantly improving computational efficiency. 2. Virtual particle and symbol particle technology: Virtual particle and symbol particles are introduced in boundary processing to effectively simulate the repulsive effect of solid boundary on fluid, reduce boundary effect and prevent non-physical penetration of fluid particles. 3. Stability and accuracy analysis: The stability evaluation equation is introduced to ensure the simulation stability, and the accuracy of simulation results is improved through numerical integration solution. Based on these innovations, the LSPH-SSPH algorithm provides more efficient and accurate simulation results when dealing with complex hydrodynamics problems.

This technology has a wide range of practical applications. Specifically, in the metal casting industry, it can be used to simulate the heat transfer process of molten metal at different casting scales, optimize casting process parameters, improve casting quality, and reduce energy consumption and cost. For example, accurately simulating the heat conduction process can determine the optimal pouring temperature and cooling time. In addition, in the field of plastic injection molding, it can be used to simulate the heat transfer of plastic melt in the mold, helping to design more reasonable mold structures and injection molding processes. For example, analyzing temperature distribution and adjusting injection speed and pressure can reduce product defects. Finally, in terms of heat dissipation design for electronic devices, this technology can simulate the heat transfer during the operation of electronic components, providing a basis for optimizing heat dissipation solutions. For example, designing more efficient heat sinks or improving the internal layout of equipment can enhance its stability and lifespan.

## 2 Related work

As China’s manufacturing industry gradually moves towards high-end and intelligent manufacturing, more researchers are paying attention to the numerical heat conduction processes in device manufacturing. To address the incompressibility in SPH fluid simulation, Ren proposed a novel incompressible SPH solver based on deformation gradient to directly measure fluid compressibility. The results showed that the algorithm was suitable for single fluid and multi-fluid simulations, significantly improving the visual effect of mixed model simulations, and supporting artistic control [6]. J. Jang proposed a real-time fluid tactile rendering technique to enhance the realism of fluid simulation and user interaction. This method calculated the pressure field on the surface of the virtual hand and transmitted it to users through an ultrasonic aerial tactile display. The results showed that this technology effectively achieved fluid tactile rendering in various scenarios [7]. S. Liu introduced the SIMPLE method into SPH to iteratively solve the incompressibility and viscosity problems caused by pressure and shear interference in simulating viscous incompressible SPH fluids. The results showed that this method not only produced true viscous behavior, but also better preserved surface details when simulating incompressible fluids [8]. Y. Zhang used the SPH to explore the dynamic temperature characteristics of cathode micro protrusions under electron emission. This method introduced a material constitutive model and state equation, considering the coupling effects of heat conduction, Joule heating, Nottingham effect, and electron emission. This method could effectively simulate and analyze the dynamic temperature characteristics of micro-protrusions under various conditions, providing reference for insulation design and state evaluation of high-voltage vacuum systems [9]. J. Chang proposed an integrated temperature sensor based on the p-GaN/AlGaN/GaN hetero-structure for chip temperature detection. This sensor utilized the opposite temperature dependence of 2DEG resistors and PiN diodes to achieve high-precision temperature detection. The results showed that under 10V power supply, the sensor had a maximum sensitivity of 19.7 mV/°C between 25°C and 300°C [10].

To address the temperature rise during normal operation of electrical equipment, E. Fjeld used the IEC 60890 standard and simplified heat conduction calculation to quickly and roughly estimate the temperature of key components in air insulated switch-gear. The results showed that this method could reduce the number of prototype and test cycles required during development, although accuracy was affected by power input and metal cooling components [11]. S. Zandi used COMSOL multi-physics field simulation software for 3D simulation analysis to optimize the performance of CZTSSe thin film solar cells. The results showed that after adding Mo(S,Se)2 layers, the open circuit voltage Voc was improved (from 0.46V to 0.513V), which was consistent with the efficiency in IBM. At the same time, the thermal distribution map of the battery was obtained, providing important reference for the thermal management of the battery [12]. X. Yang proposed an optimized temperature-dependent cauer-type insulated gate bipolar transistor module thermal network for monitoring the junction temperature of power devices in high power density and high reliability power converters. This method effectively improved prediction accuracy and significantly reduced FEM simulation time [13]. To address the serious challenges of thermal management, S. S. Salvi conducted a critical review of research literature on heat transfer in the field of 3D IC technology. The results showed that thermal modeling, thermoelectric collaborative design, and thermal management were key research areas. Key literature in recent years was summarized. It was expected that this review article helps researchers in academia and industry understand the latest developments in the field of 3D IC heat transfer [14]. A. Akbari proposed a multi-physics field heat transfer simulation method based on highly parallel finite element method to efficiently simulate heat transfer in liver radio-frequency ablation prediction. After developing a strong coupling method based on Message Passing Interface (MPI) and testing it on cluster nodes, the results showed that the method exhibited good scalability on up to 1024 cores [15]. A. Abbas Khan proposed the Cattaneo-Christov heat flux theory and similar transformations for the heat transfer of second-order fluids through exponentially stretched surfaces in porous media. The results showed that increasing the thermal relaxation parameter and solute relaxation time parameter reduced the thermal and concentration distribution, respectively. The Matlab method could solve the non-linear ordinary differential Eq [16].

Although the above studies have made significant progress in their respective fields, there are still some common shortcomings. For example, some studies lack accuracy in dealing with complex boundary conditions and require more refined boundary processing methods. Meanwhile, the computational efficiency of large-scale simulation tasks still needs to be improved, especially when dealing with multi-physics field coupling problems. Therefore, this study constructs a new numerical heat transfer simulation system based on the Lagrangian particle mathematical model and the SSPH algorithm.

## 3 Numerical heat transfer simulation system based on LSPH-SSPH algorithm

### 3.1 Construction of Lagrangian particle mathematical model

**Fig 1:**
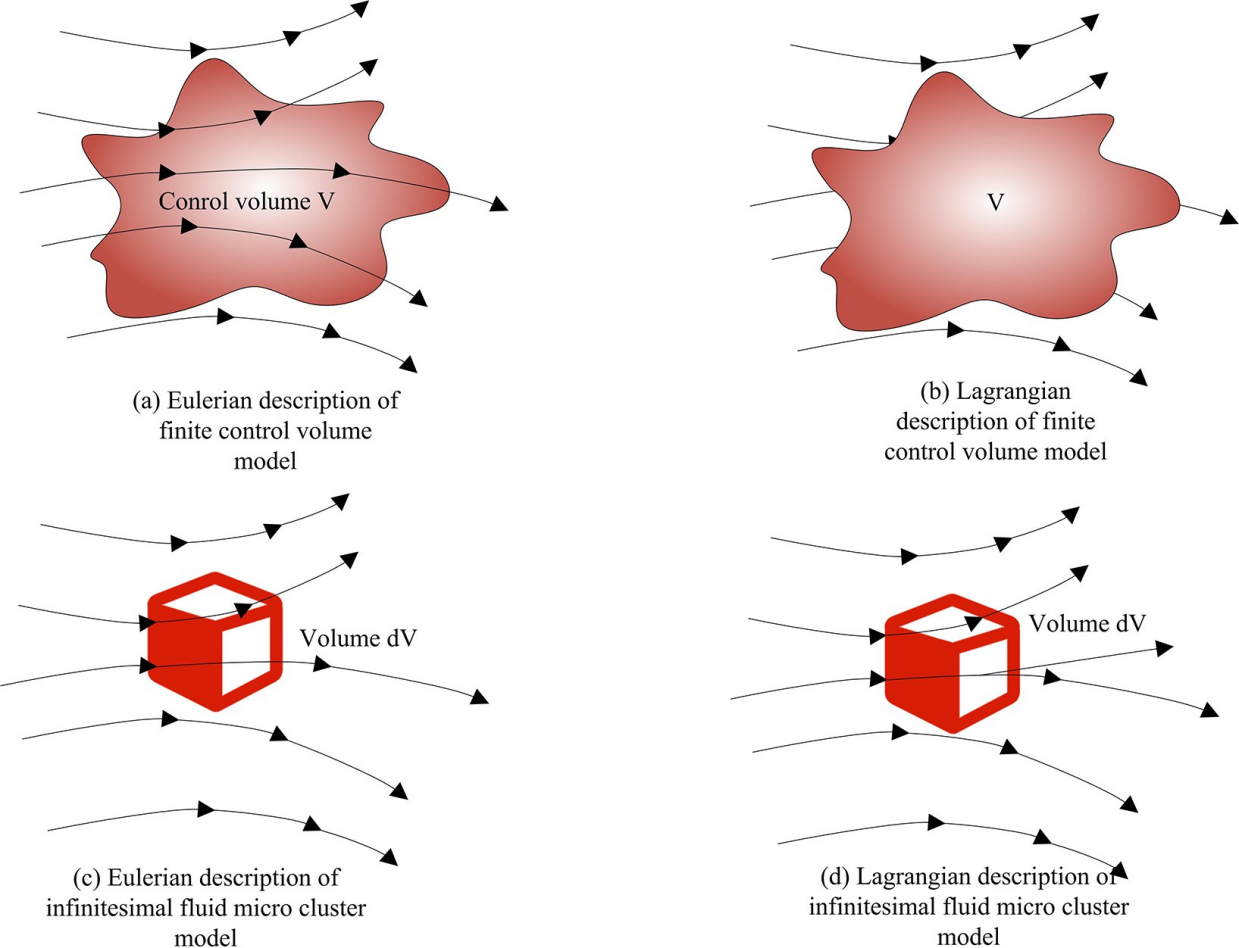
The finite control volume model and the infinitesimal fluid micro cluster model.

Fluid is a special state of matter that is fundamentally different from solids. During the motion, each part has the same velocity and exhibits rigidity. Fluid has fluidity, and the velocities of different parts are different, making it easily affected by compression and viscosity [17]. Fluid is composed of countless molecules that constantly move in space without a fixed equilibrium position. In fluid mechanics, two basic models are introduced to describe the motion of fluids, namely the finite control volume model and the infinitesimal fluid micro cluster model [18,19]. The finite control volume model focuses on the motion of a fixed region in the fluid, while the infinitesimal fluid micro cluster model focuses on the variation of a small region in the fluid over time. These two models are described using Eulerian and Lagrangian methods, respectively. Eulerian describes the position changes of fluids in space, while Lagrangian describes the trajectory of fluid micro clusters over time, as shown in Fig 1.

In fluid mechanics, the finite control volume model is used to analyze fluid motion in a flow field. As shown in Fig 1, the model defines a closed volume V and a closed control surface S within a finite region of the flow field. In the Eulerian multi-phase flow mixing model, the mathematical description of the control equation is usually in the form of partial differential equations, which is used to describe the spatial and temporal changes of fluid and particulate matter. The conservation equations of the fluid and particulate matter are shown in Eq (1).


$$\{\begin{array}{c} \frac{\partial \rho }{\partial t}+\nabla \cdot (\rho u)=0\\ \frac{\partial \rho_{p}}{\partial t}+\nabla \cdot (\rho_{p}v)=0 \end{array}$$ (1)

**Fig 2:**
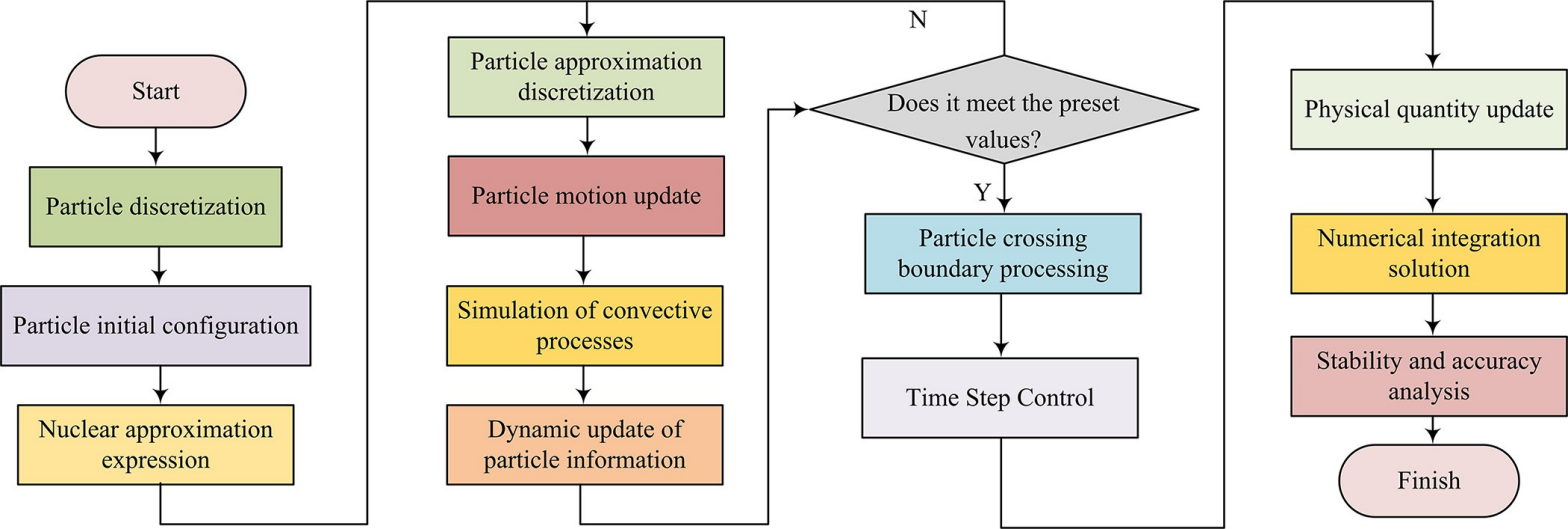
Basic steps of the mathematical model of the particles.

In Eq (1), *ρ* signifies the fluid density. *t* signifies the time. ∇⋅(*ρ***u**) signifies the divergence of the dot product of fluid density and velocity vectors, where **u** is the velocity vector of the fluid. ∇⋅(*ρ*_*p*_**v**) signifies the divergence of the dot product of the density and velocity vectors of particulate matter. **v** is the velocity vector of particulate matter. The finite control volume model focuses on the fluid within a limited area of the control volume itself, rather than viewing the entire flow field globally. The basic principles of physics can be applied to a finite controlled body to directly obtain the fluid equation in integral form. By manipulating the superposition equations of these integral forms, partial differential equations can be indirectly obtained. The motion and interaction of particulate matter in fluids have significant impacts on fluid flow characteristics [20,21]. To more accurately describe and predict the motion patterns of particulate matter in fluids, a Lagrangian particle mathematical model is established to analyze the thermal conduction interaction between different phase substances. In the field of numerical simulation, Lagrange particle mathematical models are commonly used to track the motion of specific particles or points in fluids or solid media. The basic steps are displayed in Fig 2 [22,23].

First, particles are discretized. In this process, the continuous computational region is discretized by a finite number of N particles. For any particle, it contains mass, density, velocity, small temperature, and other related physical properties. The second step is particle configuration initialization. Initially, particles are evenly distributed at equal distances under the Cartesian grid points. The mass of particles depends on the initial density distribution of the discrete medium and the volume partitioning form adopted, as shown in Eq (2).


$$m=\rho_{0}V$$ (2)

In Eq (2), *ρ*_0_ is the initial density distribution. In the uniform distribution, the expression for particle volume *V* is shown in Eq (3).


$$V=\Delta x^{d}$$ (3)

In Eq (3), *d* is the spatial dimension. The third step is to calculate the kernel approximation expression. For any physical quantity function (f) at particle *x*, it is shown in Eq (4).


$$<\nabla f>=\int f(x^{\prime })\nabla W(x-x^{\prime },h)dx^{\prime }-f_{i}\int \nabla W(x-x^{\prime },h)dx^{\prime }$$ (4)

In Eq (4), <∇*f*> is the approximate gradient value of the physical quantity function *f* at particle *x*. In the Lagrangian particle mathematical model, it is usually not possible to directly obtain the exact gradient of a continuous function at a certain point, so approximate methods need to be used for calculation. *f*(*x*′) signifies the value of the physical quantity function *f* at position *x*′. In integration, *x*′ represents all possible positions within the integration region. ∇*W*(*x*−*x*′,*h*) is the gradient of the smooth kernel function *W*, where *x*−*x*′ represents the distance between two points. *h* is the width parameter of the smooth kernel. The fourth step is to discretize the particle approximation method, using the particle approximation method to discretize the integration term in the kernel approximation expression. Continuous integration is transformed into the summation form of interactions between particles. The fifth step is to update the particle motion. During the simulation process, the position of each particle is updated based on its velocity, and particles move with their own velocity. The sixth step is to simulate the convection process. When simulating the convection process, the particle velocity is updated based on the physical model and force field. According to Newton’s second law, the acceleration caused by external or internal forces is shown in Eq (5).


$$\{\begin{array}{c} a_{i}=\frac{F_{i}}{m_{i}}=\frac{1}{m_{i}}\sum_{j\neq i}F_{ij}+F_{ext,i}\\ v_{i}(t+\Delta t)=v_{i}(t)+a_{i}\cdot \Delta t \end{array}$$ (5)

In Eq (5), *a*_*i*_ is the acceleration. *F*_*i*_ signifies the total force acting on the *i*-th particle (including internal forces such as forces caused by pressure gradients and external forces). *m*_*i*_ is the particle mass. *F*_*ij*_ represents the interaction force between the *i*-th particle and the *j*-th particle. *F*_*ext*,*i*_ is the externally applied force. The seventh step is to dynamically update particle information, which needs to be dynamically detected and updated at each simulated time step. Particle deletion and addition technology is used to manage particle collections, ensuring the accuracy and efficiency of simulations. The eighth step is to handle the particle crossing the boundary. When the particle moves and crosses the set exit boundary, it will be removed from the simulation. When the particle reaches the entrance boundary, a new particle is added to the simulation to maintain the stability of the particle count and the continuity of the simulation. The ninth step is to control the time step size. During the simulation process, the time step size is selected based on the Courant-Friedrichs-Lewy (CFL) condition, as shown in Eq (6).


$$\Delta time\leq \frac{C_{CFL}}{c_{\max}+|v_{\max}|}\cdot \min_{\mathrm{cells}}(\frac{\Delta x}{\left|\nabla v\right|})$$ (6)

In Eq (6), *C*_*CFL*_ is the CFL number. *c*_max_ is the maximum sound speed. |*v*_max_| signifies the maximum flow rate. Δ*x* is the grid size. ∇*v* is the maximum value of the velocity gradient. The tenth step is to update the physical quantities. Based on the physical model and interactions between particles, the density, temperature, and other physical properties of each particle are updated. The eleventh step is to perform numerical integration solution. In the simulation, the numerical integration method is used to solve the interaction between particles, as shown in Eq (7).


$$F_{ij}=-m_{i}m_{j}[\frac{p_{i}+p_{j}}{\rho_{i}\rho_{j}}+\Pi_{ij}]\nabla W(r_{i}-r_{j},h)$$ (7)

In Eq (7), *p*_*i*_, *p*_*j*_ represent the pressure of particles. *ρ*_*i*_, *ρ*_*j*_ are the density. Π_*ij*_ is the artificial viscosity term. The twelfth step is to conduct stability and accuracy analysis. It is necessary to conduct stability and accuracy analysis on the numerical methods used to ensure the reliability and effectiveness of simulation results.

### 3.2 SPH numerical heat transfer simulation algorithm and its improvement

A SPH numerical heat transfer simulation algorithm is constructed based on the Lagrangian particle mathematical model. It mainly includes 8 steps. Step 1 is to particle the problem domain. If the problem is not represented in particle form, a series of discretely distributed particles are used to represent the problem domain. Particles carry various physical properties of continuous media in the problem domain, such as mass, density, velocity, stress, etc. Step 2 selects a smooth function, which uses a Gaussian smooth function to define the range and intensity of particle interactions, as shown in Eq (8) [24].


$$W(r,h)=\alpha \exp(-\frac{r^{2}}{2h^{2}})$$ (8)

In Eq (8), the smooth function *W*(*r*,*h*) is configured as a Gaussian function, which is a common kernel function used for particle interaction in the SPH method. The exponential part of the function $\exp(-\frac{r^{2}}{2h^{2}})$ defines the decay mode of interaction between particles, where *r* represents the distance between particles. *h* is the smooth length of the core, which determines the range of the function. The Gaussian function is chosen as the smooth function, mainly because it has good mathematical properties, such as analytic, local, and can decay quickly, which helps to reduce the computational amount and improve the computational efficiency. Moreover, Gaussian functions can provide smooth and continuous interpolation in physical simulations, which is particularly important for simulating hydrodynamic problems. Step 3 is the kernel approximation of the field function, where the value of any point is approximated by the weighted average of the field function values corresponding to all particles in its support domain, as shown in Eq (9) [25].


$$\left(u(x)\approx \sum_{j=1}^{N}m_{j}\frac{u_{j}}{\rho_{j}}W(x-x_{j},h)\right)$$ (9)

**Fig 3:**
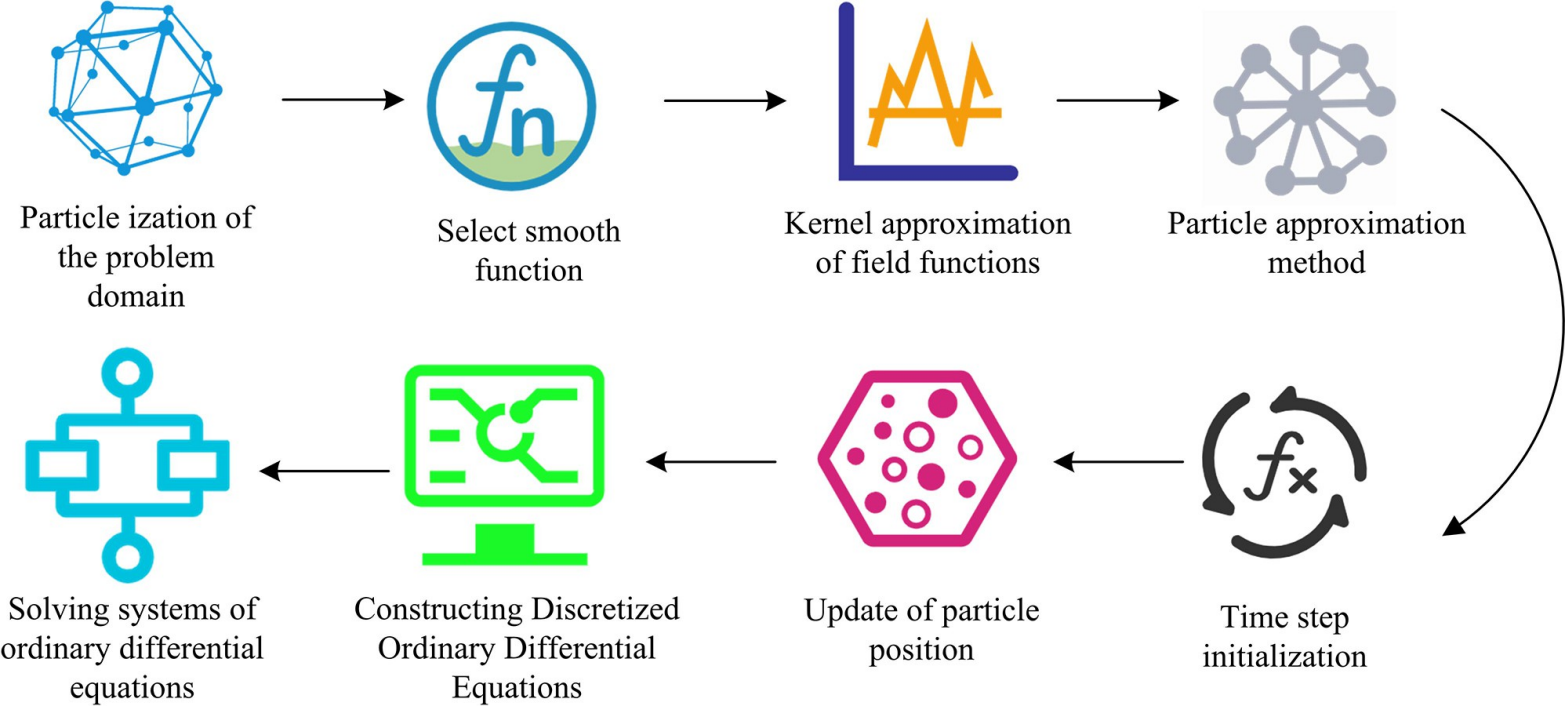
Basic flow diagram of the SPH algorithm.

In Eq (9), **x** is the position vector where the field function value needs to be calculated. *m*_*j*_ is the mass of particle *j*. *u*_*j*_ is the field function value on particle *j*. *ρ*_*j*_ is the density of particle *j*. Step 4 takes the particle approximation method to transform the integral form of the kernel approximation equation into the summation form of the interaction between particles. Step 5 initializes the time step and sets the initial conditions for simulation, including the initial position and velocity of particles. Step 6 updates the particle position. At each time step, the position of particles is updated based on its current position and velocity. Step 7 constructs a discretized ordinary differential equation. The particle approximation method is applied to all partial differential equation systems, such as continuity equations, momentum equations, etc. A series of discretized ordinary differential equations are obtained to depict the variation of particle field variables over time. Finally, the ordinary differential equation system is solved using the explicit integration method. After iterative calculation, the variation values of all particle field variables over time are obtained, thereby simulating the dynamic behavior of the entire system. The basic flowchart of the SPH algorithm is shown in Fig 3.

The SPH method is based on the meshless nature of particles, which requires special methods in boundary processing to ensure the accuracy and stability. To prevent fluid particles from penetrating the wall non physically, a series of virtual particles are arranged near the boundary to apply a reaction force. The repulsive force model is shown in Eq (10) [26].


$$\{\begin{array}{c} \{[{\left(\frac{r_{0}}{r_{ij}}\right)}^{a}-{\left(\frac{r_{0}}{r_{ij}}\right)}^{b}]\frac{x_{ij}}{r_{ij}^{2}},\;\frac{r_{0}}{r_{ij}}\leq 1;\\ 0,\;\frac{r_{0}}{r_{ij}}>1 \end{array}$$ (10)

**Fig 4:**
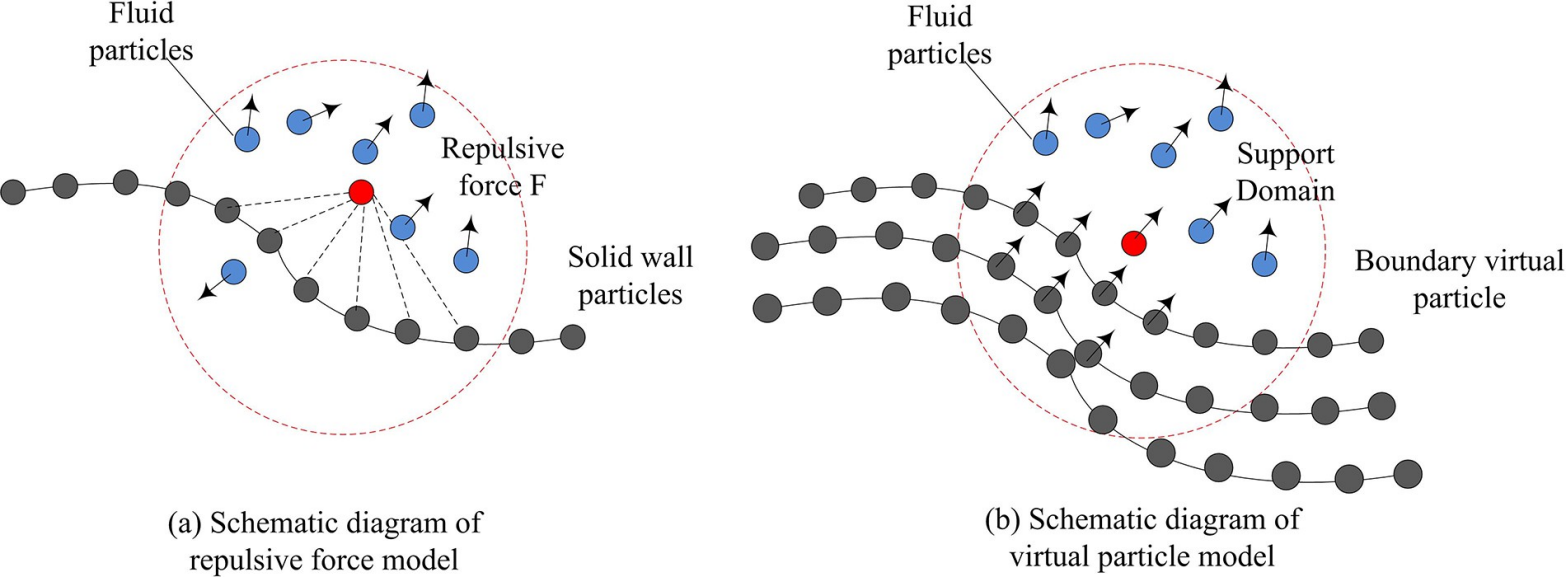
Numerical heat transfer simulation by SPH.

In Eq (10), *r*_*ij*_ signifies the distance between particles *i* and *j*. *r*_0_ is the radius of action or influence of the repulsive force. It defines the range of repulsive forces between particles. *a* and *b* are parameters of the repulsive force model, used to control the shape and strength of the repulsive force as it varies with distance. These parameters affect the stability and accuracy of the simulation. ${\left(\frac{r_{0}}{r_{ij}}\right)}^{a}$ and ${\left(\frac{r_{0}}{r_{ij}}\right)}^{b}$ terms are key components of the repulsive force model, which calculate the magnitude of the repulsive force based on the distance between particles. When the distance between particles approaches *r*_0_, the repulsive force rapidly increases to prevent particles from penetrating the boundary. $\frac{x_{ij}}{r_{ij}^{2}}$ is the component of the unit direction vector between particles (i) and (j), which is used to determine the direction of repulsion. It ensures that the repulsive force always points away from the boundary. The results of the improved virtual particle method for SPH numerical heat transfer simulation are shown in Fig 4.

In numerical heat transfer simulation, some virtual particles interact with neighboring fluid particles, generating repulsive forces to ensure that the fluid particles maintain an appropriate distance from the boundary and prevent non-physical penetration phenomena. During the simulation process, boundary virtual particles participate in solving the conservation equation of fluid particles, which effectively obtains solid wall boundary conditions. In addition, when dealing with kernel function integrals containing gradients, the boundary residual term is introduced into the partial integration process to further improve the simulation accuracy. Especially, by arranging three layers of virtual particles at the solid wall, a wider support domain is provided for fluid particles, significantly reducing the boundary effects of the SPH method and making numerical heat transfer simulations more accurate and reliable.

### 3.3 SSPH algorithm process and module design of numerical heat transfer simulation system

**Fig 5:**
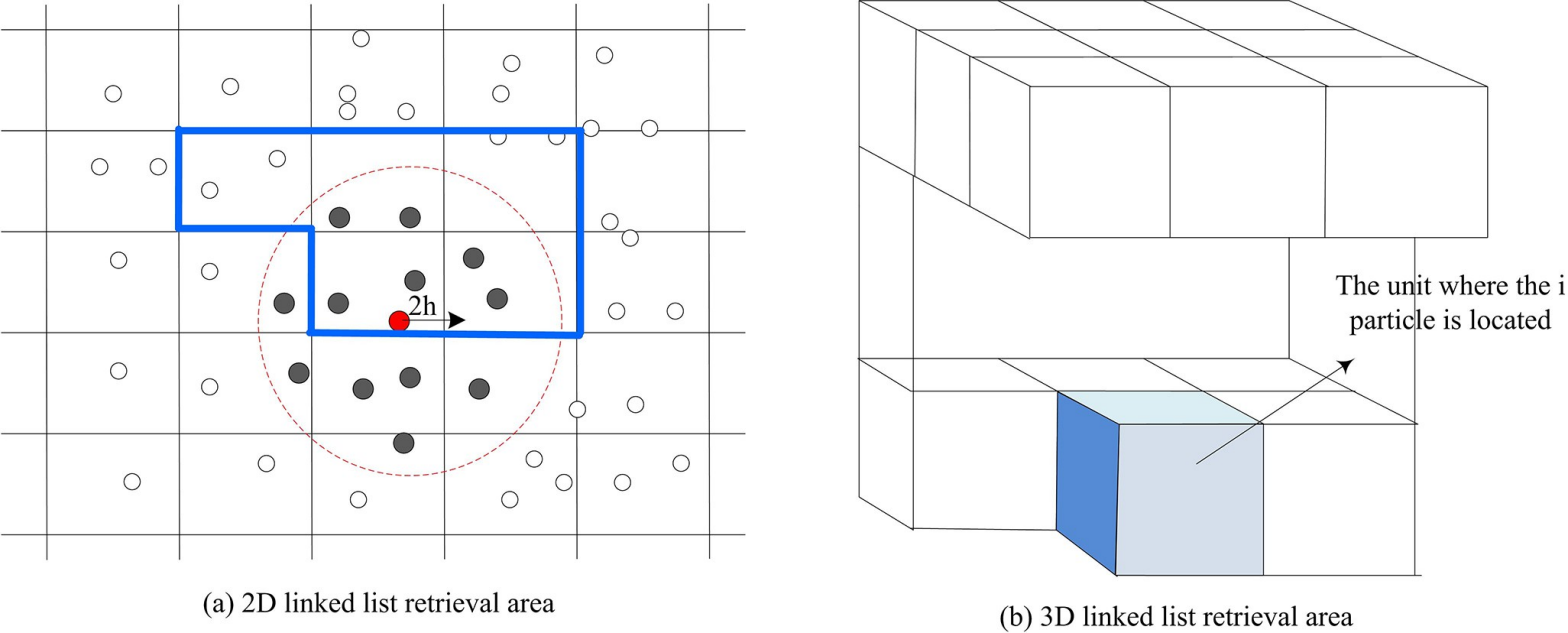
Numerical heat transfer simulation by SPH.

A major drawback of SPH numerical heat transfer simulation is that traditional neighborhood search methods (such as brute force search) can significantly reduce computational efficiency when dealing with a large number of particles, especially when frequently updating particle positions and interactions. Therefore, the linked list search method is used as an improvement strategy, dividing the problem domain into grids that match the size of the particle support domain. It is possible to quickly locate the neighborhood of particles and significantly reduce unnecessary calculations. The linked list search method is an effective method for quickly locating particle neighborhoods, which divides the entire problem domain into a series of orthogonal grids [27]. The size of these grids is carefully set to match the size of the particle support domain to ensure the search accuracy and efficiency. Taking the cubic spline kernel function as an example, due to its support domain size of 2h, the spatial size of the grid elements is also correspondingly set to 2h, as shown in Fig 5.

In two-dimensional space, when using a cubic spline function, the potential search range of each neighboring particle initially appears to be 9 grid units, forming a 9x4h^2^ area. However, due to the symmetry of interactions between particles, an optimized search strategy is used to reduce unnecessary calculations [28,29]. Specifically, when particle j is within the support domain of particle i, the nuclear functional values between them only need to be calculated once, as the force exerted by particle i on particle j is the same. In addition, traditional SPH numerical heat transfer simulations may introduce non-physical penetration phenomena when dealing with boundary conditions, which affects the simulation accuracy and stability. In addition, traditional boundary treatment methods may lead to boundary effects and affect the accuracy of simulation results. Therefore, symbolic particles are introduced as an improved method to simulate the repulsive effect of solid boundaries on fluids, reduce boundary effects, and prevent non-physical penetration of fluid particles. In terms of substance, symbol particles are assigned opposite symbols and corresponding physical properties to fluid particles, such as density and pressure. These symbol particles are placed near the solid boundary to simulate the repulsive effect of the solid boundary on the fluid. The repulsive force between the symbol particles and the fluid particles is shown in Eq (11) [30,31].


$$F_{\mathrm{repulsion}}(r_{ij})=4\epsilon [{\left(\frac{\sigma }{r_{ij}}\right)}^{12}-{\left(\frac{\sigma }{r_{ij}}\right)}^{6}]\frac{r_{ij}}{r_{ij}^{2}}$$ (11)

**Fig 6:**
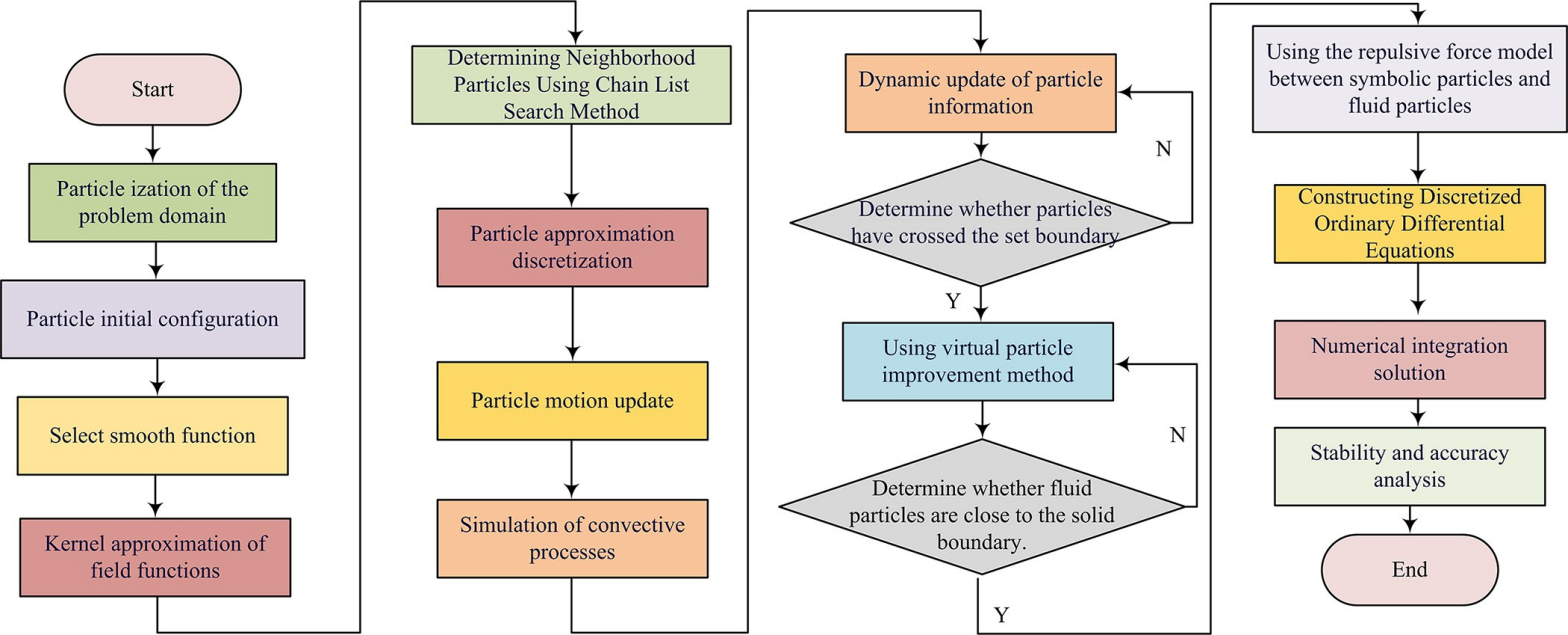
Schematic diagram of the SSPH numerical heat transfer simulation algorithm.

In Eq (11), *F*_repulsion_ (*r*_*ij*_) is the repulsive force between particles (i) and (j), and *r*_*ij*_ is the distance between (i) and (j). *σ* is the depth of the potential well (related to the strength of repulsive force). When fluid particles approach the solid boundary, symbol particles generate a repulsive force to ensure that the fluid particles remain within the physically permissible range, thereby preventing the non physical penetration phenomena. Therefore, the SSPH numerical heat transfer simulation algorithm is constructed. The algorithm steps are shown in Fig 6 [32].

Step 1 is to particle the problem domain, which is represented by a series of discretely distributed particles. Step 2 is the initial configuration of particles, which are uniformly distributed at equal distances on the Cartesian grid and assigned initial positions and velocities. Step 3 is to select a smooth function, using a Gaussian smooth function to define the range and intensity of particle interactions. Step 4 is to approximate the field function, which approximates the field function value at any point by the weighted average of the field function values corresponding to all particles in its support domain. Step 5 uses the linked list search method to determine neighboring particles and divides the problem domain into grids that match the size of the particle support domain. The linked list search method is used to quickly locate particle neighborhoods and reduce unnecessary calculations. Step 6 adopts the particle approximation method to discretize the integral term in the kernel approximation expression. The discretization of the integral term in the nuclear approximation expression is transformed into the summation form of the interaction between particles, as shown in Eq (12).


$$\nabla^{2}\phi (x_{i})\approx \sum_{j\in N(i)}\frac{m_{j}}{\rho_{j}}(\phi_{j}-\phi_{i})\nabla W_{ij}$$ (12)

In Eq (12), *N*(i) signifies the set of neighboring particles of particle *i*. *ρ*_*j*_ signifies the particle density. ∇*W*_*ij*_ signifies the gradient of the kernel function *W* with respect to **r**_*ij*_ = **x**_*j*_−**x**_*i*_. Specifically, the kernel approximation uses a smoothing function (kernel function) to give different weights to each neighbor particle, which usually decreases as the inter-particle distance increases. The kernel function affects the accuracy and stability of the approximation. The integral term in the kernel approximation expression is discretized into a summation form of the inter-particle interactions, which simplifies the calculation and improves the numerical stability. Step 7 is particle motion update, updating the position of each particle based on its current position and velocity. Step 8 simulates the convection process, update the particle velocity based on the physical model and force field, and calculate the acceleration. Step 9 is to dynamically update particle information, detecting and updating particle information dynamically at each time step. Step 10 is boundary processing, determining whether the particle has crossed the set boundary. If it is an exit boundary, the particle is deleted. If it is an entry boundary, a new particle is added. Step 11 uses the virtual particle improvement method to arrange virtual particles near the boundary and calculates their repulsive force with fluid particles to prevent non-physical penetration. The virtual particle boundary condition is shown in Eq (13).


$$F_{\mathrm{repulsion}}=k\cdot \sum_{j\in \mathrm{boundary}\;\mathrm{particles}}\frac{r_{ij}}{{\left\|r_{ij}\right\|}^{3}}$$ (13)

In Eq (13), *k* is the repulsion coefficient. **r**_*ij*_ is the distance vector between the real particle and the nearest virtual particle. Step 12 is symbol particle processing boundary to determine whether the fluid particles are close to the solid boundary. If so, the repulsive force model between symbol particles and fluid particles is used to prevent fluid particles from penetrating the boundary. Step 13 constructs a discretized ordinary differential equation. The particle approximation method is applied to all partial differential equation systems to obtain discretized forms of ordinary differential equations. Step 14 is a numerical integration solution, which applies the explicit integration method to solve the ordinary differential equation system and obtains the variation of particle field variables over time. Step 15 is stability and accuracy analysis. The numerical method used for stability and accuracy analysis ensures the reliability and effectiveness of simulation results. The stability evaluation is shown in Eq (14).


$$\Delta t\leq \frac{h}{\max|v|+C\sqrt{\frac{k_{B}T}{m}}}$$ (14)

**Fig 7:**
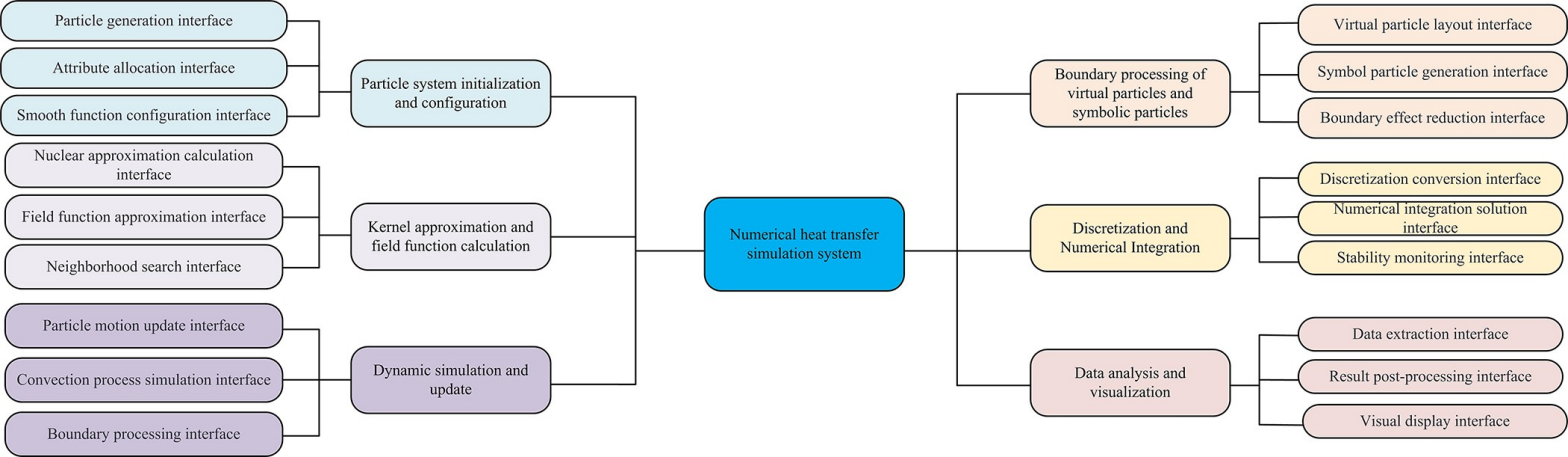
The LSPH-SSPH heat transfer simulation system.

In Eq (14), Δ*t* is the time step size. *C* is a constant related to the model. *k*_*B*_ signifies the Boltzmann constant. *T* signifies the system temperature. *m* signifies the particle mass. This equation is used to ensure the simulation stability. Therefore, the LSPH-SSPH heat transfer simulation system is constructed. Its main functional modules are shown in Fig 7.

The LSPH-SSPH heat transfer simulation system is a highly integrated simulation platform that covers the complete process from particle system initialization to data visualization analysis. The system first initializes the particle system through particle generation and attribute allocation interfaces, and configures smooth functions to accurately simulate particle interactions. Subsequently, based on kernel approximation and field function calculation modules, the system can efficiently calculate field function values at any position. The dynamic simulation and update module updates the particle state in real-time, accurately simulates the convection process, and processes boundary conditions. In response to boundary effects, the virtual particle and symbol particle boundary processing module effectively reduces non-physical penetration phenomena. The discretization and numerical integration module ensures the stability and accuracy of the simulation. Finally, the data analysis and visualization module extracts key data, performs post-processing, and presents simulation results visually through a visualization interface.

## 4 Performance testing and application experiments of numerical heat transfer simulation system

### 4.1 Performance testing of LSPH-SSPH numerical heat transfer simulation system

To improve the accuracy of heat transfer simulation process, the LSPH-SSPH numerical heat transfer simulation system is designed based on Lagrangian particle mathematical model and improved SPH algorithm. To fully verify the comprehensive performance of the system, six experimental groups are set up, with detailed settings of particle number, heat transfer model complexity, boundary condition type, fluid type, and heat source distribution. The complexity of the heat transfer model is changed by adjusting the hyper-parameter settings of the system. The experimental setting is displayed in Table 1.

**Table 1:** Experimental setting of performance test of LSPH-SSPH simulation system.

Experiment group no	Particle count	Heat transfer model complexity	Boundary condition type	Fluid type	Heat source distribution
1	10,000	Low	Static	Liquid	Uniform distribution
2	50,000	Medium	Static	Liquid	Non-uniform distribution
3	100,000	High	Dynamic	Gas	Uniform distribution
4	200,000	Medium	Dynamic	Gas	Non-uniform distribution
5	500,000	High	Complex (Multiple boundaries)	Mixed fluid	Complex distribution
6	1,000,000	Extremely high	Complex (Multiple boundaries)	Mixed fluid	Extremely complex distribution

In order to conduct performance testing on the LSPH-SSPH simulation system, an experiment is conducted on the Windows 10 platform using java language. The system is evaluated based on simulation accuracy (percentage error from actual experimental or theoretical results), simulation time (total time required to complete simulation/ms), CPU usage (average CPU usage during simulation/%), memory usage (maximum memory usage during simulation/MB), and boundary processing effect (quantitative evaluation of the degree of non-physical penetration phenomenon). Table 2 presents the experimental results.

**Table 2:** Performance test results of the LSPH-SSPH simulation system.

Experimental group number	Simulation accuracy (error%)	Simulation time (ms)	CPU usage rate (%)	Memory usage (MB)	Boundary processing effect (Non-physical penetration level)
1	2.5	120	45	500	9.5
2	2.2	300	60	1,000	9.8
3	2.1	450	60	1,500	9.2
4	2.8	500	60	2,000	9.5
5	2.5	500	61	3,500	9.8
6	2.2	500	63	4,000	9.2

**Fig 8:**
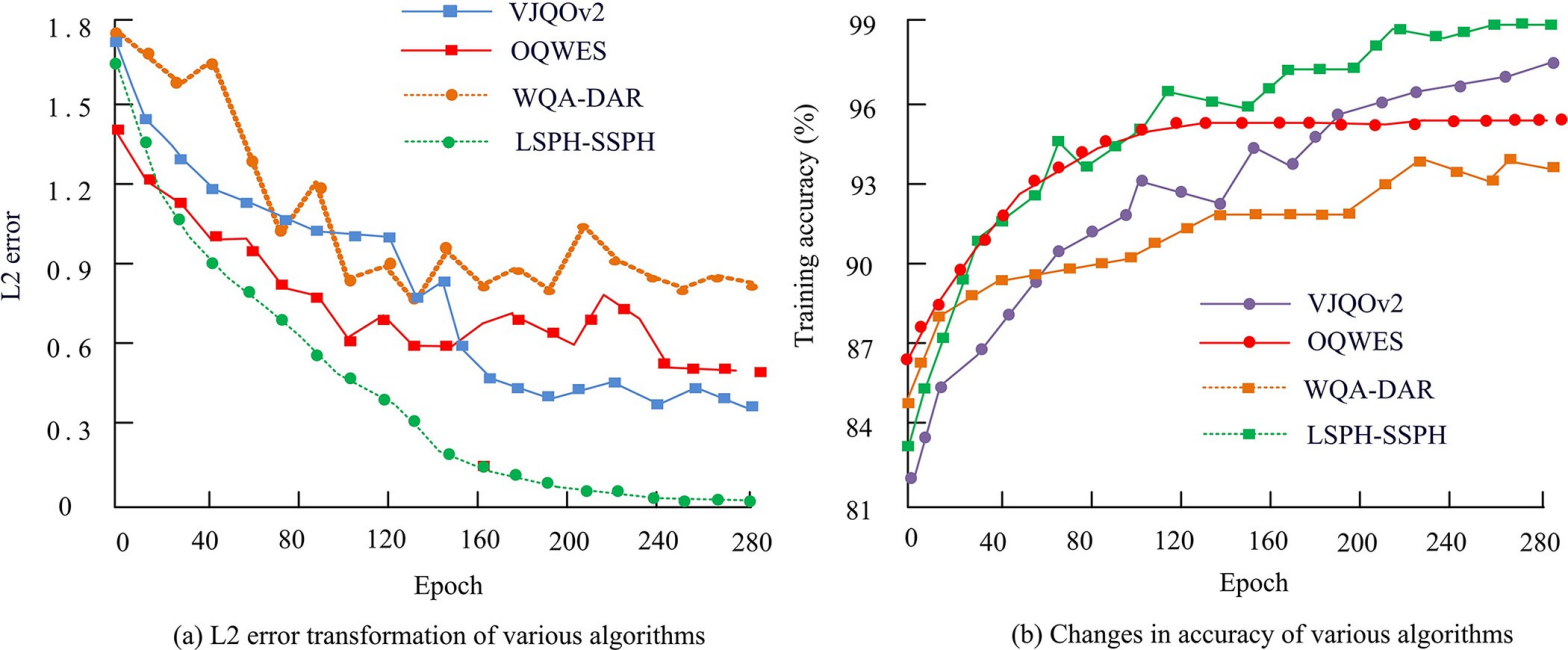
The accuracy and L2 error of each system during the training process.

According to the experimental data in Table 2, the performance test results of the LSPH-SSPH simulation system showed that the simulation accuracy remained between 2.1% and 2.8%, with a *p*-value of less than 0.05, indicating statistical significance. The standard deviation was 0.3%, indicating that the system had high simulation accuracy and good stability. There was no significant linear relationship between simulation time and the number of experimental groups (*p*>0.05). As the number of experimental groups increased, the simulation time increased, which may be related to the increase in simulation complexity. The CPU usage and memory usage increased with the increase of experimental complexity. CPU usage increased from 45% to 63% (*p*<0.05), and memory usage increased from 500MB to 4000MB (*p*<0.05), indicating an increased demand for resources when processing larger scale simulations. The score of the boundary treatment effect fluctuated between 9.2 and 9.8, with an average score of 9.5 (*p*<0.05), indicating that the boundary treatment technique was effective, which effectively controlled non-physical infiltration phenomena. Overall, the LSPH-SSPH simulation system performs well in simulation accuracy, resource utilization, and boundary handling, making it suitable for high-precision complex simulation tasks. To further validate its superiority, the WQA-DAR simulation system proposed in [33] and the Optimized Quantum Wave Energy Simulation System proposed in [34] (OQWES) and the method proposed in [35] (Versatile Joint Quantum Optimization Version 2 Simulation System, VJQOv2) are compared with the LSPH-SSPH presented in this study. The accuracy of each system during training is assessed by comparing the simulated temperature distributions, heat flux density, and differences between known solutions or experimental data. The error value of the system is calculated from the L2 error. The experimental results are shown in Fig 8.

**Fig 9:**
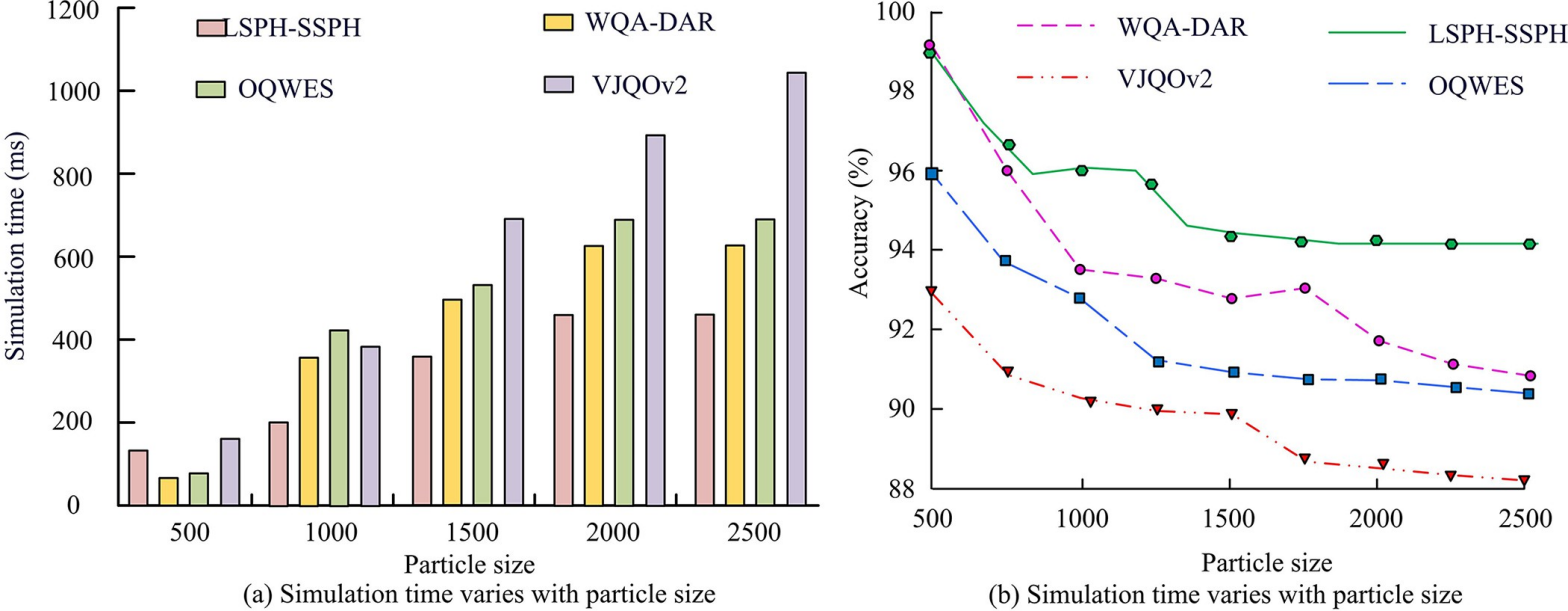
Time consumption transformation and accuracy change of different particle sizes.

In Fig 8A, all systems exhibited a rapid decrease in L2 error, indicating that all systems had good convergence performance. However, the proposed LSPH-SSPH system was particularly outstanding. Its L2 error rapidly decreased from the beginning of training and converged to an extremely low level of 0.02 after only 200 iterations, fully proving the efficiency and accuracy of the system. In contrast, the L2 error convergence values of WQA-DAR, OQWES, and VJQOv2 systems were 0.81, 0.54, and 0.34, respectively, indicating a certain performance gap. In Fig 8B, the accuracy improvement was similar, but the LSPH-SSPH system still performed well, with an accuracy of 99.1% after 240 iterations, far exceeding the other three systems. The outstanding performance of LSPH-SSPH system is mainly due to its unique algorithm design. The system uses a mathematical model of Lagrangian particles to perform a series of precise steps, including discretization of particle problem domains, initialization of particle configuration, calculation of kernel approximation expressions, discretization of particle approximation methods, updating particle motion, simulating convection processes, dynamically updating particle information, handling particle boundary crossings, controlling time steps, updating physical quantities, numerical integration solutions, and stability and accuracy analysis. At the same time, the introduced linked table search method can quickly locate particle neighborhoods, reduce unnecessary calculations, and avoid the ineffective calculations of traditional neighborhood search methods when dealing with a large number of particles. Virtual particles are used to improve boundary handling, prevent non-physical infiltration, and enhance the accuracy and stability of simulations. In contrast, WQA-DAR, OQWES, and VJQOv2 systems may have deficiencies in particle modeling, boundary processing, and other aspects, resulting in higher L2 error convergence values and relatively lower accuracy [33–35]. In addition, the study also compares the simulation time and accuracy changes of different systems for different particle sizes, as illustrated in Fig 9.

From Fig 9A, the simulation time of each system increased to varying degrees with the increase of particle size. Among them, VJQOv2 performed the worst, and its simulation time showed a linear increase with the increase of particle size. The proposed LSPH-SSPH system tended to stabilize when the particle size exceeded 2000 and eventually converged to 434ms. From Fig 9B, the LSPH-SSPH system also performed the best on accuracy variation. When the particle size reached 1500, its accuracy converged to 95.2%. The accuracy of WQA-DAR, OQWES, and VJQOv2 systems converged to 91.5%, 91.3%, and 88.1%.

### 4.2 Experimental results on the applicability of LSPH-SSPH numerical heat transfer simulation system

**Fig 10:**
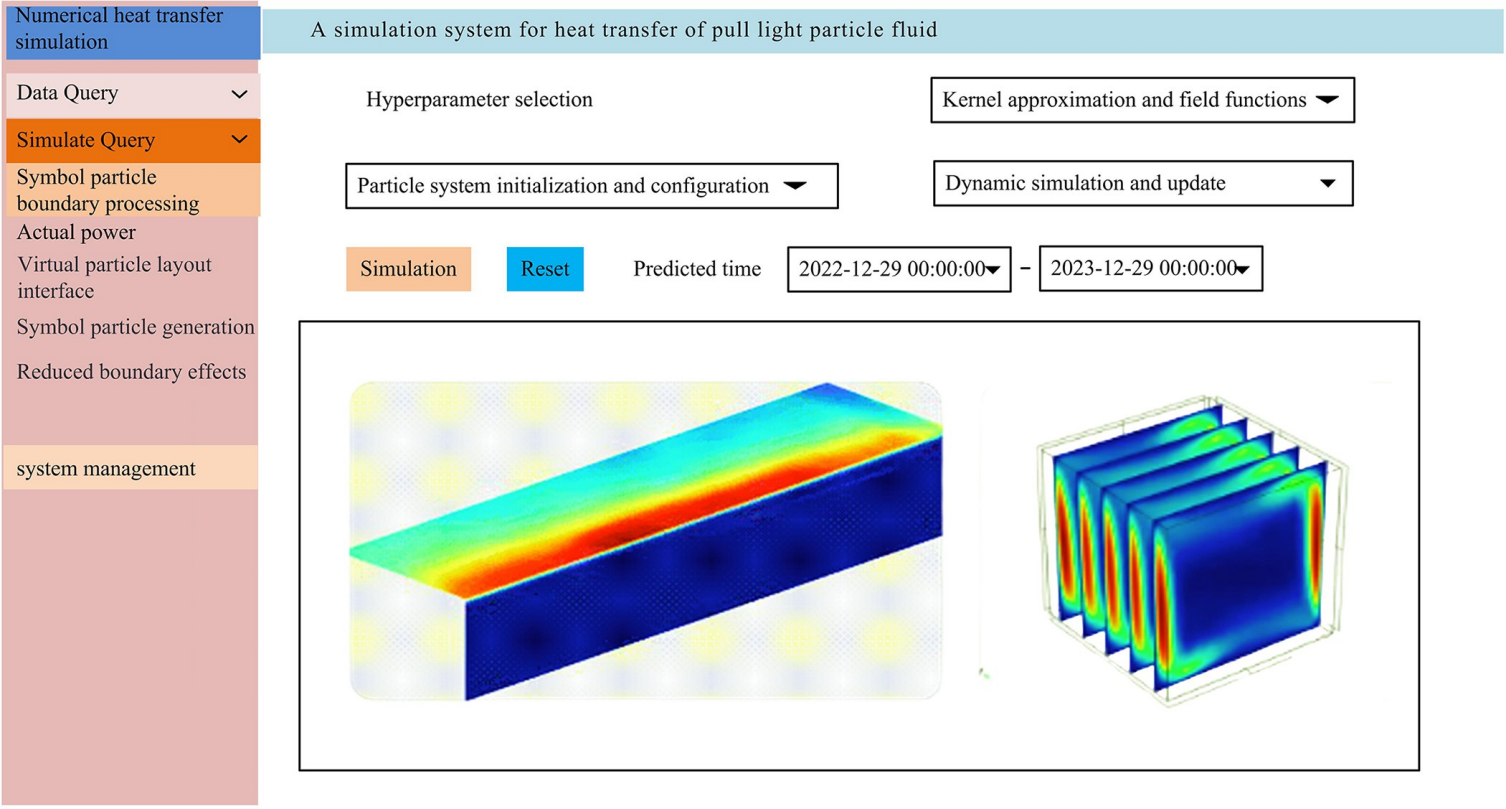
The actual prediction interface diagram of the system.

The study fully verifies the excellent performance of the LSPH-SSPH numerical heat transfer simulation system. To further verify that the system also performs similarly well in practical applications, the study implements the system on the Windows 11 platform, and sets the smoothing length to 0.05m, the initial particle spacing to 0.01 micrometers, the time step to 0.1 milliseconds, the symbol particle density to 100 times the fluid density, and the pressure to between 10^5 and 10^7 pascals. The neighborhood search grid size of the linked list search method is 0.05ms. The actual prediction interface of the system is shown in Fig 10.

**Fig 11:**
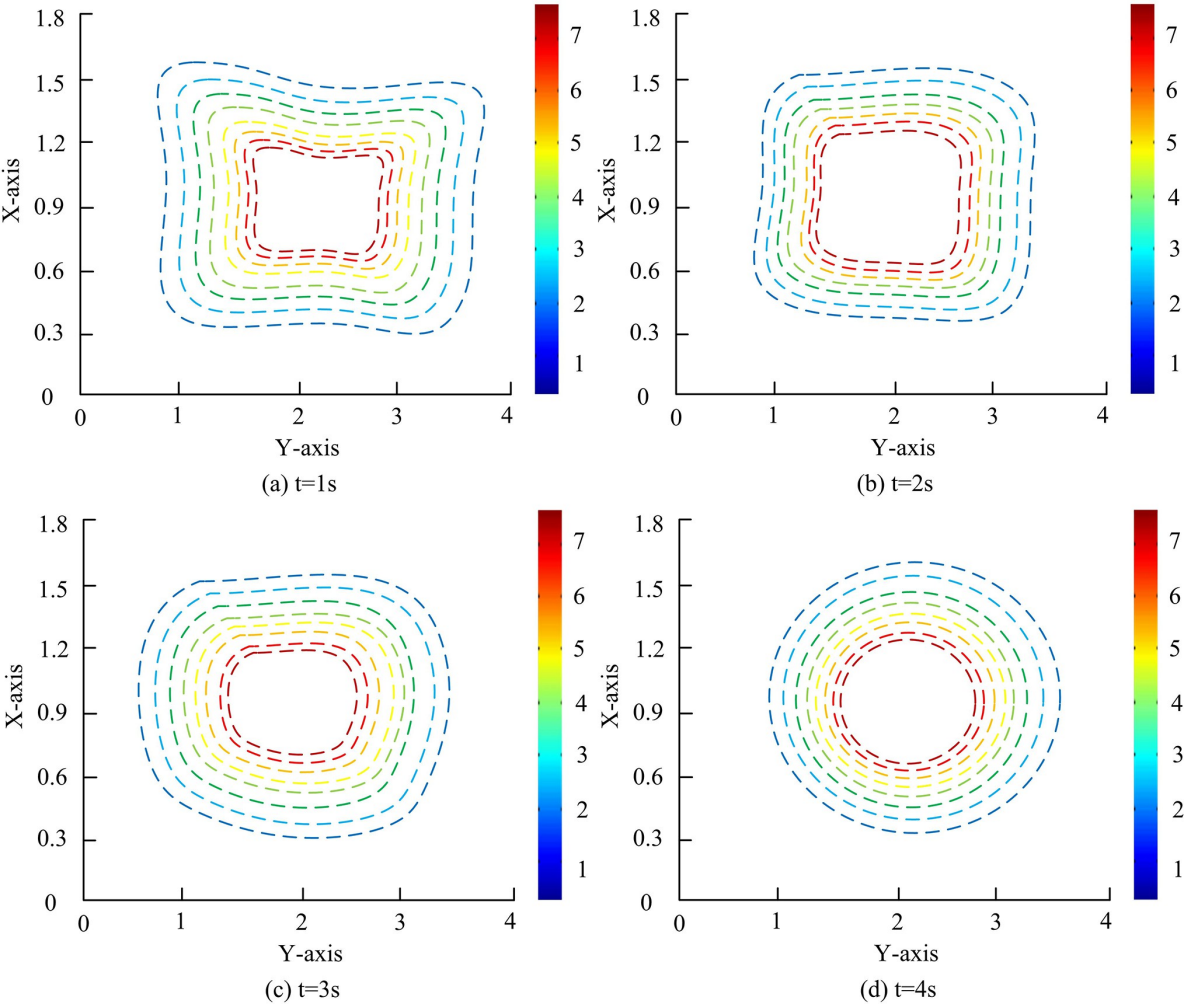
The temperature contour plot changes at different periods.

From Fig 10, the system contains multiple modules, including particle system initialization and configuration, kernel approximation and field function calculation, dynamic simulation and update, virtual particle and symbolic particle boundary processing, discretization and numerical integration, and data analysis and visualization. On the upper part of the image system, multiple hyper-parameters of the system can be selected, and below the system is the visualization area of simulation results, which clearly displays the numerical heat transfer simulation of the system. A numerical heat transfer simulation is conducted on a certain device model using this system. The temperature contour simulation results at different time periods are shown in Fig 11.

**Fig 12:**
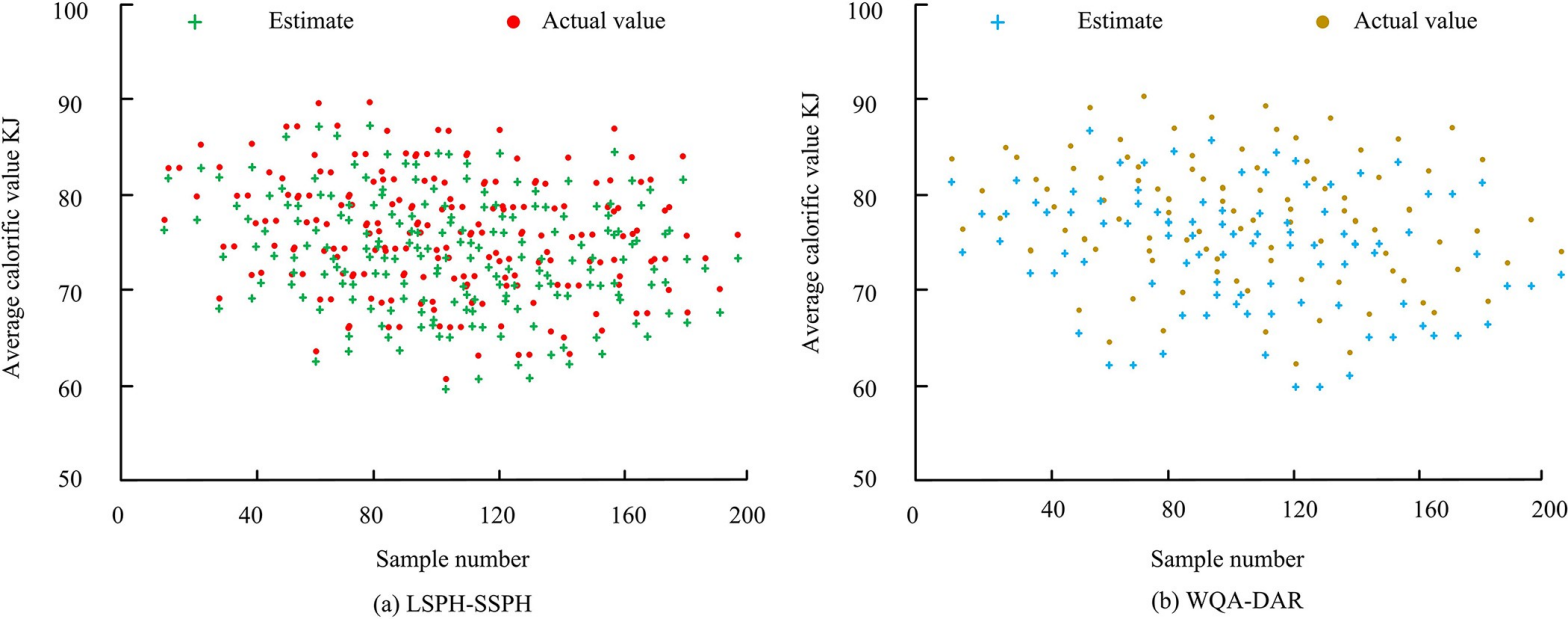
Prediction error of the average calorific value of two numerical heat transfer simulation systems.

From Fig 11, as time increased, the high-temperature area gradually expanded, while the amplitude of temperature fluctuations also gradually decreased. This observation is perfectly consistent with the law of heat conduction, further verifying the simulation accuracy. It is worth noting that the smooth transition of the contour lines indicates that the results calculated using the SSPH method does not exhibit non-physical oscillations, ensuring the authenticity and reliability of simulation results. In addition, the heat value contour gradually changed from an initial irregular shape to a concentric circular shape, which may be due to the combined effects of uniform heat diffusion and boundary effects during heat conduction. In addition, thermal conduction simulation experiments are conducted on 200 different devices. The WQA-DAR system with better performance is introduced for comparison, as illustrated in Fig 12.

**Fig 13:**
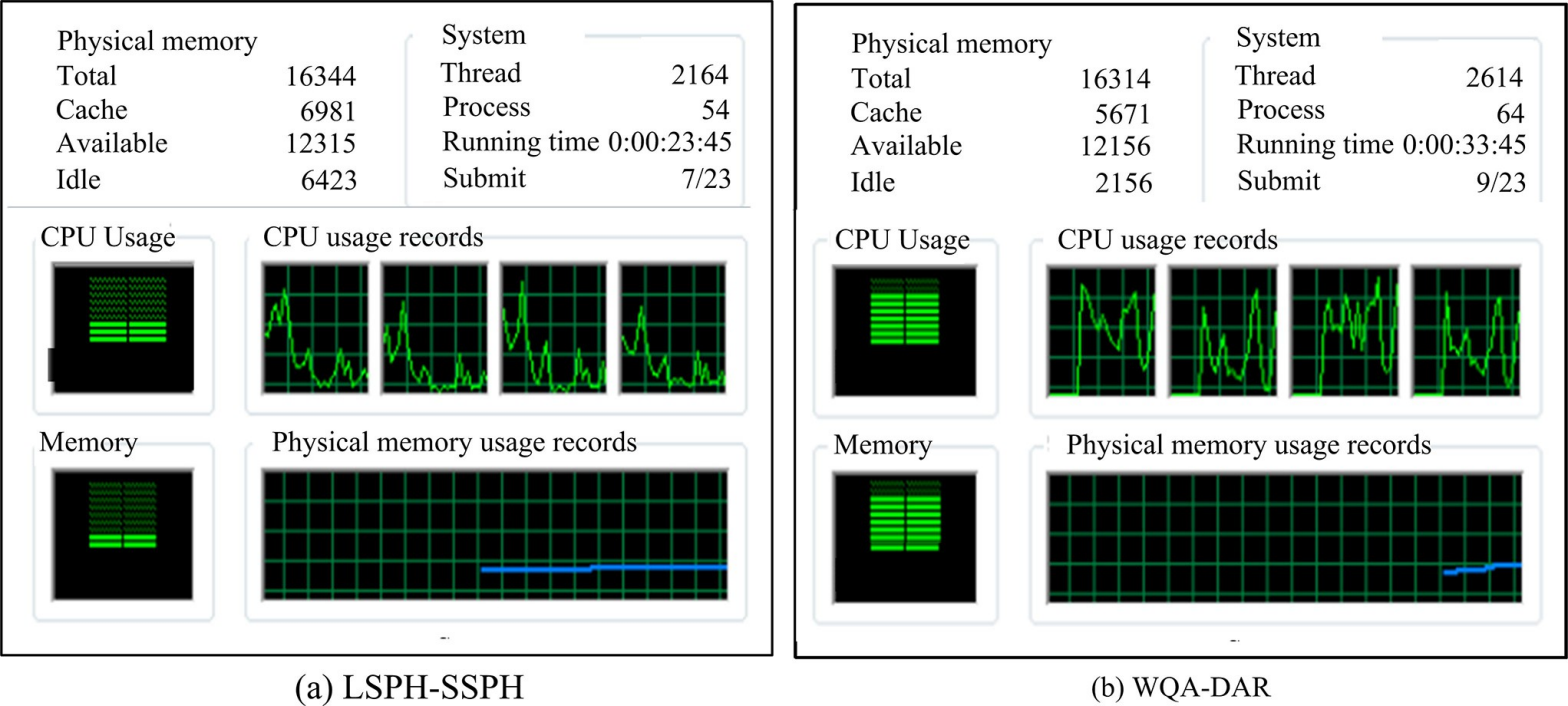
Comparison of resource consumption of two numerical heat transfer simulation systems.

From Fig 12A, the LSPH-SSPH numerical heat transfer simulation system accurately predicted the heating values of 200 different devices, with an error of no more than 1%. From Fig 12B, the WQA-DAR system had an average prediction error of over 3% for the heating values of 200 different devices. In addition, the study also records the resource consumption of the two systems during the simulation process, as presented in Fig 13.

**Fig 14:**
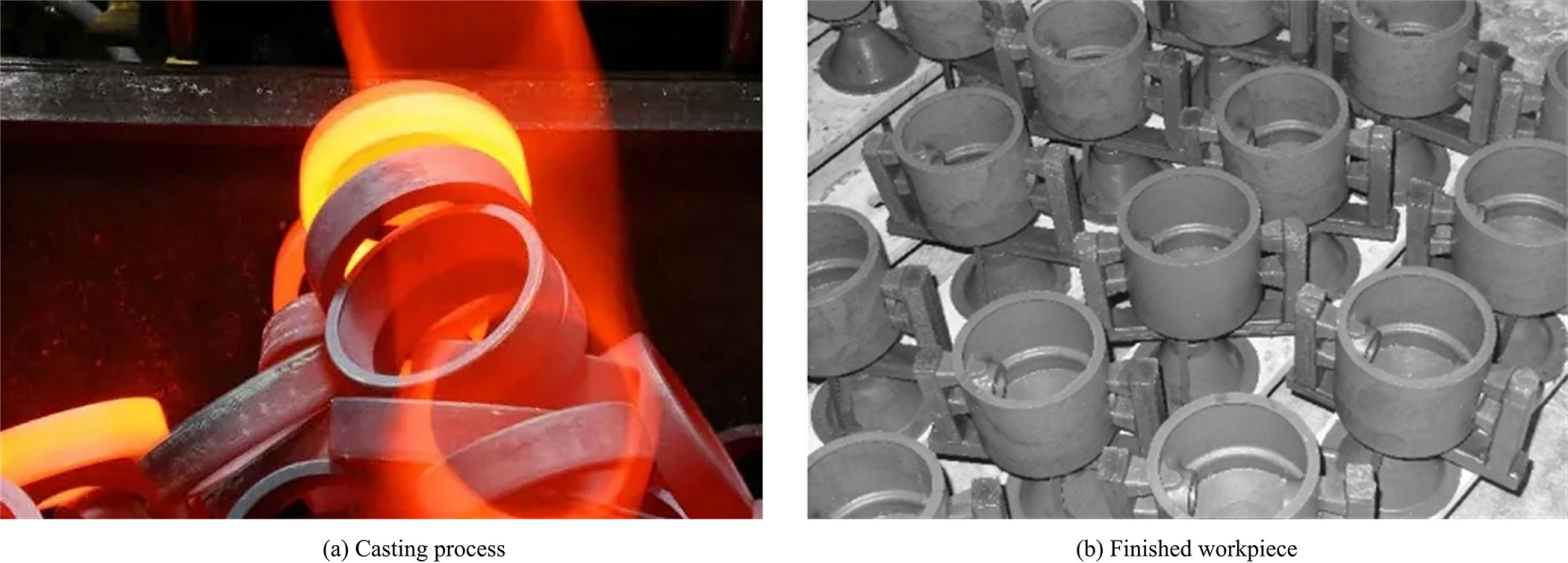
Actual workpiece casting effect.

From Fig 13A, the LSPH-SSPH numerical heat conduction model performed excellently in terms of computational performance. The average CPU usage of this model during running time was only 23%, which means that it can run efficiently at lower processor loads. Meanwhile, its physical memory usage rate was controlled at a relatively low level, only at 18%. In contrast, the WQA-DAR numerical heat conduction model in Fig 13B showed a higher resource usage rate, with an average CPU usage rate of 82% and a physical memory usage rate of 77%. This indicates that under the same conditions, the LSPH-SSPH model has greater advantages in computational efficiency. Finally, the LSPH-SSPH system is applied in the actual casting process, and the results are shown in Fig 14.

Fig 14A shows a molten metal casting with flames surrounding it, indicating that the casting process is in progress. Fig 14B shows some finished products with a gray tone, which is a cooled casting. This contrast illustrates that the casting process is a high to low temperature process, changing from a hot liquid state to a solid state. This application further confirms the validity and usefulness of the LSPH-SSPH model in a practical production environment. This efficient computational model can help manufacturers optimize production processes, improve production efficiency, and reduce energy consumption and cost. The LSPH-SSPH heat transfer simulation system has good scalability under different casting scales and conditions for small and large castings. In large-scale repeated simulations, various heat transfer manufacturing scenarios and corresponding materials such as metal casting and plastic injection molding are mainly included. The linked list search method divides the problem domain into grids that match the particle support domain, quickly locates particle neighborhoods, reduces unnecessary calculations, and adapts to a large number of particles. Under different temperature ranges and boundary shapes, virtual particle and symbolic particle boundary processing modules can effectively handle various boundary situations and prevent non-physical penetration phenomena. At the same time, the modular design of the system can add or adjust functional modules according to specific needs to adapt to different simulation scales and complex conditions, ensuring the wide applicability of the system.

## 5 Conclusions

This study successfully designed and implemented an LSPH-SSPH numerical heat transfer simulation system based on Lagrangian particle mathematical model and improved SPH algorithm. The experimental results showed that the LSPH-SSPH system exhibited excellent performance in simulation tasks of different complexities, with a simulation accuracy of up to 99.1%, a stable simulation time of 434ms, and CPU and memory usage rates as low as 23% and 18%, respectively, significantly better than comparison systems. In addition, the system introduced the virtual particle and symbol particle strategy when dealing with boundary conditions, effectively reducing non-physical penetration phenomena and improving the reliability of simulation results. The simulation error of the LSPH-SSPH simulation system fluctuated between 2.1% and 2.8%, indicating high accuracy and relative stability of the system. The memory usage increased from 500MB to 4000MB. Finally, the performance score of boundary processing fluctuated between 9.8 and 9.2. Overall, the LSPH-SSPH simulation system was less affected by the experimental scale when dealing with complex simulation tasks, which could handle large-scale simulation tasks without losing accuracy. In summary, the LSPH-SSPH numerical heat transfer simulation system provides an efficient and accurate simulation tool for the casting industry, which helps to improve product quality and production efficiency. Although the LSPH-SSPH system performs well, there is still room for improvement in computational efficiency when dealing with extremely complex scenarios and large-scale simulation tasks.

## Supporting information

S1 Data set Minimal data set definition. (DOC)
