# Supplementary material for: Numerical heat transfer simulation system based on Lagrangian particle mathematical model and SSPH algorithm
Source: PLoS One. 2024 Dec 31;19(12):e0313250. doi: 10.1371/journal.pone.0313250 (PMC11687755; doi:10.1371/journal.pone.0313250)
Supplement: S1 Data set — (DOC) [file pone.0313250.s001.doc]

**Table 1 Figure 8 The minimum dataset**

| L2 error transformation of various algorithms | | | | |
| --- | --- | --- | --- | --- |
| Epoch | VJQOv2 | OQWES | WQA-DAR | LSPH-SSPH |
| 40 | 1.2 | 1.1 | 1.6 | 1.0 |
| 80 | 1.1 | 0.8 | 1.2 | 0.6 |
| 120 | 1.0 | 0.7 | 0.9 | 0.4 |
| 160 | 0.6 | 0.6 | 0.9 | 0.2 |
| 200 | 0.4 | 0.6 | 0.9 | 0.1 |
| 240 | 0.4 | 0.6 | 0.9 | 0.1 |
| Training accuracy (%) | | | | |
| Epoch | VJQOv2 | OQWES | WQA-DAR | LSPH-SSPH |
| 40 | 86 | 91 | 88 | 91 |
| 80 | 91 | 94 | 90 | 93 |
| 120 | 92 | 95 | 91 | 96 |
| 160 | 93 | 95 | 92 | 97 |
| 200 | 96 | 95 | 92 | 97 |
| 240 | 96 | 95 | 94 | 98 |

**Table 2 Figure 9 The minimum dataset**

| Simulation time (ms) | | | | |
| --- | --- | --- | --- | --- |
| Particle size | VJQOv2 | OQWES | WQA-DAR | LSPH-SSPH |
| 500 | 190 | 100 | 90 | 170 |
| 1000 | 390 | 410 | 380 | 180 |
| 1500 | 700 | 520 | 460 | 380 |
| 2000 | 900 | 660 | 600 | 410 |
| 2500 | 1040 | 660 | 600 | 410 |
| Accuracy (%) | | | | |
| Particle size | VJQOv2 | OQWES | WQA-DAR | LSPH-SSPH |
| 500 | 93 | 96 | 99 | 99 |
| 1000 | 90 | 93 | 94 | 96 |
| 1500 | 90 | 91 | 93 | 94 |
| 2000 | 88 | 91 | 92 | 94 |
| 2500 | 88 | 91 | 91 | 94 |

**Table 3 Figure 12 The minimum dataset**

| Sample number | Estimate | Actual value |
| --- | --- | --- |
| 40 | 81.2 | 81.3 |
| 80 | 82.3 | 82.1 |
| 120 | 70.6 | 70.5 |
| 160 | 85.6 | 85.7 |
| 200 | 76.1 | 76.3 |
| Sample number | Estimate | Actual value |
| 40 | 76.6 | 78.3 |
| 80 | 80.6 | 78.6 |
| 120 | 80.3 | 81.3 |
| 160 | 90.3 | 88.1 |
| 200 | 79.6 | 82.6 |
